# Management of Dyslipidemia in Type 2 Diabetes: Recent Advances in Nonstatin Treatment

**DOI:** 10.3390/diseases6020044

**Published:** 2018-05-24

**Authors:** Kazutoshi Sugiyama, Yoshifumi Saisho

**Affiliations:** Division of Endocrinology, Metabolism, and Nephrology, Department of Internal Medicine, Keio University School of Medicine, 35 Shinanomachi, Shinjuku-ku, Tokyo 160-8582, Japan; ksugiyama@keio.jp

**Keywords:** dyslipidemia, type 2 diabetes, PCSK9, nonstatin

## Abstract

Dyslipidemia is a major risk factor for cardiovascular disease (CVD), which is the leading cause of morbidity and mortality in type 2 diabetes (T2DM). Statins have played a crucial role in its management, but residual risk remains since many patients cannot achieve their desired low-density lipoprotein cholesterol (LDL-C) level and up to 20% of patients are statin-intolerant, experiencing adverse events perceived to be caused by statins, most commonly muscle symptoms. Recently, great advances have been made in nonstatin treatment with ezetimibe, a cholesterol absorption inhibitor, and proprotein convertase subtilisin/kexin type 9 (PCSK9) monoclonal antibodies (mAbs), all showing a proven benefit with an excellent safety profile in cardiovascular outcome trials. This review summarizes the key aspects and the evolving role of these agents in the management of dyslipidemia in patients with T2DM, along with a brief introduction of novel drugs currently in development.

## 1. Introduction

Cardiovascular disease (CVD) is the leading cause of morbidity and mortality in individuals with type 2 diabetes (T2DM) [1,2]. In the Finnish study, which showed that T2DM is a coronary heart disease (CHD) equivalent, type 2 diabetic patients without prior myocardial infarction (MI) and nondiabetic patients with prior MI had a similar incidence of MI and risk of CHD death. Furthermore, when less stringent criteria for prior CHD (MI, angina pectoris, or ischemic electrocardiogram changes) were used, T2DM carried a larger risk than prior CHD [3,4]. Diabetic dyslipidemia, characterized by increased triglyceride (TG) level and decreased high-density lipoprotein cholesterol (HDL-C) level, is a major risk factor for CVD. Although low-density lipoprotein cholesterol (LDL-C) level is typically normal, small dense LDL particles, which are more atherogenic since they are more likely to undergo glycation and oxidation, are more prevalent in T2DM [5].

CARDS (Collaborative Atorvastatin Diabetes Study) [6] and the Heart Protection Study [7] have shown the efficacy of statins in diabetic patients, and in the Cholesterol Treatment Trialists’ meta-analysis of diabetic patients, statins reduced major vascular events by 21% and all-cause mortality by 9% for each 38.7 mg/dL (1.0 mmol/L) reduction in LDL-C [8]. Major guidelines recommend statins for the treatment of dyslipidemia in T2DM [2,9,10,11,12], but even with high-dose statins, 12.7% and 40.4% of patients do not achieve LDL-C levels below 100 mg/dL and 70 mg/dL, respectively [13]. Moreover, in clinical practice, statin-associated muscle symptoms occur in up to 20% of patients and contribute to their discontinuation [14]. New treatment strategies are needed, and this review focuses on recent advances in nonstatin treatment, with special attention to proprotein convertase subtilisin/kexin type 9 (PCSK9) monoclonal antibodies (mAbs).

## 2. Search Strategy

We searched PubMed, ClinicalTrials.gov, and other sources for articles published in English between 1 January 2000 and 1 April 2018, using the search terms “dyslipidemia”, “type 2 diabetes”, “PCSK9”, “evolocumab”, and “alirocumab”. We also searched the reference lists of articles identified by this search strategy along with manually selected articles known to the authors.

## 3. Nonstatin Lipid-Lowering Therapies

### 3.1. Ezetimibe

Ezetimibe reduces cholesterol absorption by inhibiting Niemann-Pick C1-Like 1 (NPC1L1) protein in the small intestine and hepatocytes [15,16]. In the IMPROVE-IT (Improved Reduction of outcomes: Vytorin Efficacy International Trial) [17], the first trial to show an improvement in cardiovascular (CV) outcomes with the addition of a nonstatin drug to a statin, 18,144 patients who had been hospitalized for an acute coronary syndrome (ACS) within the preceding 10 days were randomized to simvastatin–ezetimibe combination therapy or simvastatin monotherapy. With a median follow-up of 6 years, the addition of ezetimibe to simvastatin reduced LDL-C by 16 mg/dL and resulted in a 6.4% reduction (32.7% vs. 34.7%; hazard ratio (HR) 0.936; 95% confidence interval (CI) 0.89–0.99; *p* = 0.016) in the primary endpoint, which was a composite of CV death, MI, unstable angina requiring hospitalization, coronary revascularization, or stroke, compared to simvastatin monotherapy (Table 1). There were no differences in adverse events, including muscle-related events.

Of the study subjects, 4933 (27%) had diabetes at baseline, and compared to patients without diabetes, ezetimibe was associated with an enhanced benefit ((HR 0.86; 95% CI 0.78–0.94 for patients with diabetes) vs. (HR 0.98; 95% CI 0.92–1.04 for patients without diabetes); *p* value = 0.023 for interaction) in the primary endpoint with similar safety outcomes [21]. In addition, in a prespecified analysis which compared outcomes stratified by achieved LDL-C level at 1 month, the adjusted HRs for the primary endpoint favored lower achieved LDL-C groups (HRs of 1.0, 0.82, 0.80, and 0.79 for LDL-C >70, 50–69, 30–49, and <30 mg/dL, respectively; p for trend <0.001) without an increase in adverse events [22]. These results suggest the benefit of the addition of ezetimibe in diabetic patients after an ACS and perhaps also in patients with stable clinical atherosclerotic cardiovascular disease (ASCVD).

### 3.2. PCSK9 Inhibitors

In 2003, gain-of-function mutations in PCSK9 were reported as a cause of hypercholesterolemia [23]. Soon after, low LDL-C in individuals with loss-of-function mutations in PCSK9 was reported [24], and a moderate lifelong reduction in LDL-C (15–28%) resulted in a substantial reduction in the incidence of CHD by 47–88% [25]. Furthermore, individuals with no circulating PCSK9 and very low LDL-C due to compound heterozygous loss-of-function mutations in PCSK9 were apparently healthy [26], making PCSK9 inhibition a very attractive target for LDL-C-lowering therapy.

Plasma LDL-C binds to LDL receptors expressed on the surface of hepatocytes and is internalized by endocytosis [27]. LDL receptors are usually recycled to the cell surface, but when PCSK9 binds with LDL receptors, LDL receptors are delivered to lysosomes for degradation, resulting in lower expression of LDL receptors and an increase in LDL-C [28]. Therapeutic approaches targeting extracellular PCSK9 (e.g., mAbs) and intracellular PCSK9 (e.g., small interfering RNAs (siRNAs)) are currently under investigation, but mAbs have been the most successful strategy thus far [29].

#### 3.2.1. Monoclonal Antibodies

Evolocumab and alirocumab, two subcutaneous agents currently available on the market, have been studied in numerous populations, including familial hypercholesterolemia, diabetes, and statin intolerance, as monotherapy and in combination with statins. In a meta-analysis of phase 2 and 3 studies, treatment with PCSK9 mAbs reduced LDL-C by 55% [30]. Moreover, OSLER (Open-Label Study of Long-Term Evaluation against LDL Cholesterol) 1 and 2 [31] and ODYSSEY LONG TERM (Long-term Safety and Tolerability of Alirocumab in High Cardiovascular Risk Patients with Hypercholesterolemia Not Adequately Controlled with Their Lipid-Modifying Therapy) [32], which were long-term studies of 1 to 1.5 years, have shown a significant reduction in CV events of roughly 50%. However, the number of CV events was small, and confirmation in trials adequately powered to examine CV outcomes are eagerly awaited. The results of FOURIER (Further Cardiovascular Outcomes Research with PCSK9 Inhibition in Subjects with Elevated Risk) [18] and ODYSSEY Outcomes [19] have been recently reported and are discussed below.

#### 3.2.2. Cardiovascular Outcomes Trial: FOURIER

FOURIER [18] included 27,564 patients with ASCVD and LDL-C ≥ 70 mg/dL who were receiving statin therapy. Patients were randomly assigned to evolocumab (140 mg every 2 weeks or 420 mg monthly) or placebo, and at 48 weeks, evolocumab reduced LDL-C by 59% compared to placebo, from a median baseline value of 92 mg/dL to 30 mg/dL. With a median follow-up of 2.2 years, evolocumab significantly reduced the primary endpoint, which was a composite of CV death, MI, stroke, hospitalization for unstable angina, or coronary revascularization, by 15% (9.8% vs. 11.3%; HR 0.85; 95% CI 0.79–0.92; *p* < 0.001), and the key secondary endpoint, which was a composite of CV death, MI, or stroke, by 20% (5.9% vs. 7.4%; HR 0.80; 95% CI 0.73–0.88; *p* < 0.001) (Table 1). Although the magnitude of the risk reduction in the primary and key secondary endpoints appeared to grow over time, there were no significant differences in CV death (HR 1.05; 95% CI 0.88–1.25) and all-cause mortality (HR 1.04; 95% CI 0.91–1.19).

Diabetes was present at baseline in 11,031 (40%) patients, and in a prespecified secondary analysis [20], similar efficacy in the primary and key secondary endpoints was observed in patients with and without diabetes. However, since patients with diabetes had a higher baseline risk, they seemed to have a greater absolute risk reduction in the primary endpoint over 3 years ((2.7%; number needed to treat 37) vs. (1.6%; number needed to treat 62); *p* = 0.60 for interaction). This benefit was driven largely by a greater absolute risk reduction in coronary revascularization, and there was no difference in the absolute risk reduction for the key secondary endpoint.

#### 3.2.3. Cardiovascular Outcomes Trial: ODYSSEY Outcomes

ODYSSEY Outcomes [19] included 18,924 patients who had been hospitalized for an ACS 1 to 12 months prior to randomization. After a run-in period of 2 to 16 weeks on high-intensity statins, patients with LDL-C ≥ 70 mg/dL, non-HDL-C ≥ 100 mg/dL, or apolipoprotein B ≥ 80 mg/dL were randomized to alirocumab (75 mg every 2 weeks) or placebo. A target LDL-C level of 25 to 50 mg/dL was specified, with up-titration of alirocumab to 150 mg every 2 weeks in patients with LDL-C ≥ 50 mg/dL and a blinded switch to placebo in patients who consistently had LDL-C < 15 mg/dL.

In the on-treatment analysis, which excluded LDL-C values after premature treatment discontinuation or blinded switch to placebo, alirocumab reduced LDL-C by 61% from a mean LDL-C of 96.4 mg/dL to 42.3 mg/dL at 1 year, and by 54.7% from a mean LDL-C of 101.4 mg/dL to 53.3 mg/dL at 4 years. With a median follow-up of 2.8 years, alirocumab significantly reduced the primary endpoint, which was a composite of CHD death, MI, ischemic stroke, or unstable angina requiring hospitalization, by 15% (9.5% vs. 11.1%; HR 0.85; 95% CI 0.78–0.93; *p* = 0.0003), and the secondary composite endpoint of all-cause death, MI, or ischemic stroke by 14% (10.3% vs. 11.9%; HR 0.86; 95% CI 0.79–0.93; *p* = 0.0003) (Table 1). Although all-cause death was significantly lower with alirocumab, there were no significant differences in CHD death and CV death.

In a prespecified secondary analysis stratified by baseline LDL-C, patients with LDL-C ≥ 100 mg/dL appeared to gain the most benefit, with reductions in the primary and secondary endpoints, although the p value for interaction was not significant. Roughly 30% of patients had diabetes and considering that diabetes is associated with higher mortality after an ACS [33], a greater absolute risk reduction might have been seen in this population, and we await the results of further analysis.

#### 3.2.4. Safety of Monoclonal Antibodies and Low LDL-C

Cholesterol is an essential component of all cell membranes and is critical to the maintenance of normal cell functions, such as gonadal hormones, adrenal function, and brain function. Therefore, theoretical concerns have been raised regarding the extremely low level of LDL-C achieved with PCSK9 mAbs [34]. Both evolocumab and alirocumab were safe and well tolerated in FOURIER [18] and ODYSSEY Outcomes [19], although longer follow-up periods are needed since both trials had a relatively short follow-up period of less than 3 years. A prespecified secondary analysis of FOURIER [35] showed a highly significant monotonic relationship between achieved LDL-C and major CV outcomes, without a significant association with safety outcomes, which is in accordance with the results of a secondary analysis from IMPROVE-IT [22]. Currently, evolocumab has been studied for up to 4 years in the open-label OSLER-1 extension study and has shown a good safety profile [36]. An open-label extension study of FOURIER is ongoing, which will investigate 6600 subjects for 5 years and will provide more information regarding its long-term safety (NCT03080935, NCT02867813). Key adverse events are briefly discussed below.

#### 3.2.5. Muscle-Related Events

In FOURIER [18], rates of muscle-related events were similar between evolocumab and placebo (5.0% vs. 4.8%, respectively). Details of ODYSSEY Outcomes [19] have not been reported yet, but in ODYSSEY LONG TERM [32], alirocumab had a higher rate of myalgia compared to placebo (5.4% vs. 2.9%, respectively).

#### 3.2.6. Injection Site Reactions

In FOURIER [18] and ODYSSEY Outcomes [19], injection site reactions were rare, but were significantly more frequent with both evolocumab (2.1% vs. 1.6%) and alirocumab (3.8% vs. 2.1%) compared to placebo.

#### 3.2.7. Antidrug Antibodies

The development of bococizumab, a humanized mAb with approximately 3% of the murine sequence remaining, was discontinued in part due to the development of a high rate of antidrug antibodies which diminished the magnitude and durability of LDL-C reduction [37]. In contrast, evolocumab and alirocumab are fully humanized mAbs, and in FOURIER [18], only 0.3% of patients developed new antidrug antibodies, and development of neutralizing antibodies did not occur in any patient. In ODYSSEY Outcomes [19], neutralizing antibodies developed in 42% and 6% of patients in the alirocumab and placebo group, respectively, and slight attenuation of LDL-C lowering over time was observed in the trial. Further analyses are needed to elucidate whether neutralizing antibodies had a negative effect or if it was mainly due to the trial design with a specified down-titration algorithm at low LDL-C levels. In a previous report of 4747 patients from 10 trials of alirocumab, neutralizing antibodies were observed in 1.3% of patients, but reductions in LDL-C were maintained over time regardless of neutralizing antibody status [38].

#### 3.2.8. Neurocognitive Events

In FOURIER [18] and ODYSSEY Outcomes [19], there were no significant differences in neurocognitive events for both evolocumab (1.6% vs. 1.5%) and alirocumab (1.5% vs. 1.8%) compared to placebo. Cognitive function was prospectively assessed in a subgroup of patients from FOURIER in EBBINGHAUS (Evaluating PCSK9 Binding Antibody Influence on Cognitive Health in High Cardiovascular Risk Subjects) [39] using the Cambridge Neuropsychological Test Automated Battery (CANTAB), a computerized cognitive assessment tool that uses touch-screen neuropsychological tests of cognition that are specifically designed to assess central nervous system disorders and cognitive function. A total of 1204 patients were followed for a median of 19 months, and there were no significant differences in the CANTAB score between patients who received evolocumab and placebo. The ongoing 5-year extension of FOURIER includes CANTAB assessments in approximately 500 patients who had also participated in EBBINGHAUS and will provide longer-term data regarding cognition. A clinical trial of alirocumab is also ongoing, with prospective CANTAB assessments in 2200 patients with a follow-up period of 2 years (NCT02957682). Lastly, a recently reported mendelian randomization study provides reassurance, as genetic variants in PCSK9 and 3-hydroxy-3-methylglutaryl-coenzyme A reductase (HMGCR, the target of statins) showed no causal effects of low LDL-C on the risk of Alzheimer’s disease, Parkinson’s disease, and dementia [40].

#### 3.2.9. New-Onset Diabetes

Meta-analyses of randomized trials have shown a dose-dependent relationship between statins and risk of incident diabetes, with a higher risk in patients receiving intensive-dose therapy compared with moderate-dose therapy (OR 1.12; 95% CI 1.04–1.22) [41,42]. Mendelian randomization studies with genetic variants in PCSK9 have also shown an increased risk of diabetes [43,44], and whether PCSK9 mAbs carry a risk of development of diabetes has been a matter of concern. In a prespecified secondary analysis of FOURIER [20], evolocumab did not increase the risk of new-onset diabetes in patients without diabetes at baseline (HR 1.05; 95% CI 0.94–1.17), including in those with prediabetes (HR 1.00; 95% CI 0.89–1.13). Levels of HbA1c and fasting plasma glucose were similar between the evolocumab and placebo groups over time in patients with diabetes, prediabetes, or normoglycemia. In ODYSSEY Outcomes [19], alirocumab did not increase the risk of new-onset diabetes in patients without diabetes at baseline (9.6% vs. 10.1%) and did not have an adverse effect of worsening diabetes or diabetic complications in patients with diabetes at baseline (18.8% vs. 21.2%).

#### 3.2.10. Cost-Effectiveness

The efficacy and safety of evolocumab and alirocumab in FOURIER [18] and ODYSSEY Outcomes [19] are very promising, but with a hefty price tag of $14,000 per year in the United States. Three cost-effectiveness analyses [45,46,47] incorporating data from FOURIER have been reported, with an incremental cost-effectiveness ratio (ICER) of $268,600 to $450,000 per quality-adjusted life-year (QALY), and a 60% to 70% reduction from current prices would be needed to achieve a societally acceptable ICER of $100,000 per QALY [48].

#### 3.2.11. Intracellular PCSK9 Inhibitors: Inclisiran

Inclisiran is a long-acting, subcutaneously delivered siRNA targeting PCSK9 messenger RNA (mRNA) [49]. It is attached to an N-acetylgalactosamine moiety, which facilitates selective uptake into liver cells via the asialoglycoprotein receptor [50]. After binding intracellularly to the RNA-induced silencing complex (RISC), it specifically cleaves mRNA encoding PCSK9 [49].

In ORION-1 [51], a phase 2 study of inclisiran, 501 patients at high risk for CVD, of which 118 (24%) patients had diabetes at baseline, with elevated LDL-C despite maximum tolerated dose of statins were randomized to receive a single dose of placebo or 200, 300, or 500 mg inclisiran or two doses (on days 1 and 90) of placebo or 100, 200, or 300 mg inclisiran. Inclisiran reduced PCSK9 and LDL-C levels in a dose-dependent manner, and at 6 months, LDL-C reductions of 27.9% to 41.9% after a single dose and 35.5% to 52.6% after two doses (*p* < 0.001 for all comparisons vs. placebo) were observed. The two-dose 300 mg inclisiran regimen produced the greatest reduction in LDL-C with a mean reduction of 64.2 mg/dL from baseline, with 48% and 66% of patients achieving LDL-C <50 mg/dL and <70 mg/dL, respectively. There seemed to be no adverse events related to inclisiran, but 5% of patients who received inclisiran experienced injection-site reactions whereas no injection-site reactions occurred in patients assigned to placebo.

Whether there is a clinically meaningful difference in intracellular and extracellular PCSK9 inhibition remains unknown. However, inclisiran has the advantage of a twice-yearly injection and a lower manufacturing cost when compared with PCSK9 mAbs, which require an injection every 2 to 4 weeks with a substantial cost burden [52]. ORION-10 (NCT03399370) and ORION-11 (NCT03400800), phase 3 studies recruiting a combined total of 3,000 patients with ASCVD and elevated LDL-C despite maximum tolerated dose of statins, are ongoing, and ORION-4 [53], a randomized CV outcome trial of inclisiran in 15,000 patients with stable ASCVD and LDL-C ≥ 100 mg/dL with a median follow-up of 5 years, is planned.

### 3.3. Bempedoic Acid

Bempedoic acid (ETC-1002) is a once-daily, orally administered prodrug that inhibits adenosine triphosphate citrate lyase (ACL), a key enzyme upstream of HMGCR involved in the synthesis of fatty acids and cholesterol [54,55]. It also activates 5′-adenosine monophosphate-activated protein kinase (AMPK), reducing the activity of acetyl-CoA carboxylase (ACC) and HMGCR, the rate-limiting enzymes of fatty acid and cholesterol synthesis, respectively [55]. The activation of AMPK also targets phosphoenolpyruvate carboxykinase and glucose-6-phosphatase, enzymes with a crucial role in gluconeogenesis and liver glucose production [55] and seems to have a favorable effect on glucose regulation in animal models [56]. In the liver, the prodrug is activated by very long-chain acyl-CoA synthetase-1 (ASCVL1), but since skeletal muscle does not express ASCVL1, it remains in its inactive form and can potentially avoid the myotoxicity associated with statins [54].

In phase 2 clinical trials, bempedoic acid has shown significant LDL-C reductions of up to 50% when combined with ezetimibe [57], and in patients with type 2 diabetes [56], it was associated with a nonsignificant reduction in fasting and postprandial glucose concentrations, along with a nonsignificant tendency of improved glycemic control in a 24-h continuous glucose monitoring assessment compared to placebo. A phase 3 CV outcome trial involving 12,600 high-risk patients who are statin intolerant is ongoing (CLEAR Outcomes; NCT02993406).

### 3.4. Fibrates

Fibrates are agonists of peroxisome proliferator-activated receptor alpha (PPARα), mediating transcription factors that control lipoprotein metabolism [58]. They improve the lipid profile of diabetic dyslipidemia by decreasing TG level and increasing HDL-C level, but recent trials have failed to show a benefit in outcomes, both with monotherapy [59] and in addition to a statin [60]. In the ACCORD (Action to Control Cardiovascular Risk in Diabetes) lipid trial [60], the addition of fenofibrate to simvastatin in high-risk patients with T2DM did not reduce the primary endpoint, which was a composite of MI, stroke, or CV death, with a mean follow-up of 4.7 years. However, in a prespecified subgroup analysis, a possible benefit for patients with both a high baseline TG level ≥ 204 mg/dL and a low baseline level of HDL-C ≤ 34 mg/dL was suggested, with similar post hoc subgroup analysis in the FIELD (Fenofibrate Intervention and Event Lowering in Diabetes) study [61]. PROMINENT (Pemafibrate to Reduce Cardiovascular OutcoMes by Reducing Triglycerides IN patiENts with diabeTes; NCT03071692), a CV outcome trial of pemafibrate is currently underway, and will investigate 10,000 patients with T2DM who have TG level ≥ 200 mg/dL and HDL-C level ≤ 40 mg/dL despite concomitant statin therapy.

### 3.5. Omega 3 Fatty Acids

Omega 3 fatty acids (eicosapentaenoic acid (EPA) and docosahexaenoic acid (DHA)) decrease TG level, but they have produced even more inconsistent results than fibrates [62]. We await the results of two ongoing CV outcome trials, REDUCE-IT (Reduction of Cardiovascular Events with Icosapent Ethyl-Intervention Trial; NCT01492361) and STRENGTH (A Long-Term Outcomes Study to Assess STatin Residual Risk Reduction With EpaNova in HiGh Cardiovascular Risk PatienTs With Hypertriglyceridemia; NCT02104817), which will evaluate the effect of omega 3 fatty acids on top of statins in high-risk patients with mixed dyslipidemia.

## 4. Therapeutic Strategies for Dyslipidemia in Patients with Type 2 Diabetes

Some guidelines recommend an LDL-C treatment target, while others recommend a specific statin intensity without an LDL-C target (Table 2) [2,9,10,11,12]. However, they all agree on statins as the first-line treatment, and problems arise when patients cannot achieve their target LDL-C level or are statin-intolerant. First and foremost, accurate identification of true statin intolerance is of vital importance, since many patients are able to tolerate statins when rechallenged. Statin-associated muscle symptoms are usually not of pharmacological origin, but rather a consequence of patient perceptions that statins can cause muscle symptoms, combined with the high background prevalence of muscle symptoms in middle-aged and elderly patients [14]. In GAUSS-3 (Goal Achievement After Utilizing an Anti-PCSK9 Antibody in Statin Intolerant Subjects 3) [63], 511 patients intolerant to multiple statins were rechallenged in a double-blinded manner with one half of the patients randomized to atorvastatin 20 mg and the other half randomized to placebo for the first 10 weeks, with subsequent crossover to the alternate treatment group. During this rechallenge phase, 26.5% of patients experienced muscle symptoms with placebo but not with atorvastatin, supporting the aforementioned notion, although true statin intolerance clearly exists in some patients as 42.6% of patients experienced muscle symptoms with atorvastatin but not with placebo.

Ezetimibe should be used in patients who fail to achieve their target LDL-C level with statins alone or are statin-intolerant in view of the benefits proven in IMPROVE-IT [17]. We prefer ezetimibe over PCSK9 mAbs due to its oral administration and low cost, and PCSK9 mAbs should be reserved for very high-risk patients with clinical ASCVD and LDL-C above their target level despite concomitant use of statins and ezetimibe. Gemfibrozil, a type of fibrate, inhibits statin glucuronidation and leads to the elevation of plasma statin concentration. Therefore, it should not be combined with statins because of an increased risk of myotoxicity [64]. In contrast, fenofibrate does not have a significant effect on statin glucuronidation, and there was no increased risk in the ACCORD lipid trial where fenofibrate–simvastatin combination therapy was investigated [60]. The addition of fenofibrate may be considered in high-risk patients with elevated TG level and low HDL-C level despite statin treatment, taking into account the suggested benefit in the subgroup analyses of ACCORD lipid [60] and FIELD [61].

## 5. Conclusions

Statins have been and will remain the cornerstone of treatment of dyslipidemia in patients with diabetes. However, residual risk remains, and nonstatin treatment with ezetimibe and PCSK9 mAbs has an evolving role with proven benefits on CV outcomes. As the most potent LDL-C-lowering agent available on the market, PCSK9 mAbs have huge expectations, but their long-term safety remains to be established, and their prices must come down for them to be cost-effective. Inclisiran would be a huge addition to our arsenal especially if their market price is much cheaper than PCSK9 mAbs. The potential of bempedoic acid to avoid the myotoxicity of statins with a favorable effect on glucose metabolism is exciting, and we eagerly await the results of CV outcome trials of fibrates and omega 3 fatty acids to further understand the role of high TG level in CVD. We have made huge progress but much remains to be done, and further advances to improve the care of our patients should be anticipated.

## Figures and Tables

**Table 1:** Cardiovascular outcome trials of nonstatin drugs.

Variable	IMPROVE-IT [17]	FOURIER [18]	ODYSSEY Outcomes [19]
No. of patients	18,144	27,564	18,924
No. of patients with diabetes	4933 (27%)	11,031 (40%) [20]	5444 (29%)
Mean age (years)	64	63	58
Clinical characteristics	ACS within 10 days	ASCVD and LDL-C ≥70 mg/dL or non-HDL-C ≥100 mg/dL on statin	ACS within 12 months; LDL-C ≥70 mg/dL or non-HDL-C ≥100 mg/dL or ApoB ≥80 mg/dL on high-intensity statin
Intervention	Simvastatin 40 mg and ezetimibe 10 mg vs. simvastatin 40 mg	Evolocumab 140 mg q 2w or 420 mg q 4w vs. placebo	Alirocumab 75–150 mg q 2w vs. placebo
Primary endpoint	CV death, MI, stroke, hospitalization for UA, coronary revascularization	CV death, MI, stroke, hospitalization for UA, coronary revascularization	CHD death, MI, ischemic stroke, hospitalization for UA
Median f/u (years)	6	2.2	2.8
Achieved LDL-C (mg/dL)	53.7 vs. 69.5	30 vs. 92	53.3 vs. 101.4
Primary endpoint	32.7% vs. 34.7%; HR 0.936 (95% CI 0.89–0.99); *p* = 0.016	9.8% vs. 11.3%; HR 0.85 (95% CI 0.79–0.92); *p* < 0.001	9.5% vs. 11.1%; HR 0.85 (95% CI 0.78–0.93); *p* = 0.0003
3-point MACE (CV death, MI, stroke)	22.2% vs. 20.4%; HR 0.90 (95% CI 0.84–0.96); *p* = 0.003	5.9% vs. 7.4%; HR 0.80 (95% CI 0.73–0.88); *p*<0.001	10.3% vs. 11.9%; HR 0.86 (95% CI 0.79–0.93); *p* = 0.0003 *
CV death	6.8% vs. 6.9%; HR 1.00 (95% CI 0.89–1.13); *p* = 1.00	1.8% vs. 1.7%; HR 1.05 (95% CI 0.88–1.25); *p* = 0.62	2.5% vs. 2.9%; HR 0.88 (95% CI 0.74–1.05); *p* = 0.15
All-cause death	15.3% vs. 15.4%; HR 0.99 (95% CI 0.91–1.07); *p* = 0.78	3.2% vs. 3.1%; HR 1.04 (95% CI 0.91–1.19); *p* = 0.54	3.5% vs. 4.1%; HR 0.85 (95% CI 0.73–0.98); *p* = 0.026
Adverse events	Similar safety in both groups	Injection-site reactions: 2.1% vs. 1.6% Neutralizing antibodies: 0% in both groups	Injection site reactions: 3.8% vs. 2.1% Neutralizing antibodies: 42% vs. 6%

ACS = acute coronary syndrome; AMI = acute myocardial infarction; ApoB = apolipoprotein B; ASCVD = atherosclerotic cardiovascular disease; CHD = coronary heart disease; CI = confidence interval; CV = cardiovascular; FOURIER = Further Cardiovascular Outcomes Research with PCSK9 Inhibition in Subjects with Elevated Risk; HR = hazard ratio; HDL-C = high-density lipoprotein cholesterol; IMPROVE-IT = Improved Reduction of outcomes: Vytorin Efficacy International Trial; LDL-C = low-density lipoprotein cholesterol; MACE = major adverse cardiovascular events; MI = myocardial infarction; UA = unstable angina; * 3-point MACE for all-cause death, MI, stroke.

**Table 2:** Recommendations for treatment of dyslipidemia in patients with T2DM.

(a) Guidelines with LDL-C treatment target recommendations				
	**ASCVD (+)**	**ASCVD (−) and Other CVD Risk Factors (+)**	**ASCVD (−) and Other CVD Risk Factors (−)**	**Nonstatin Treatment**
**2017 AACE/ACE [11]**	LDL-C < 55 mg/dL	LDL-C < 70 mg/dL	LDL-C < 100 mg/dL	If LDL-C above target, consider ezetimibe. If ASCVD (+), also consider PCSK9 mAb.
	**ASCVD (+)**	**ASCVD (−) and Other CVD Risk Factors (+)**	**ASCVD (−) and Other CVD Risk Factors (−)**	**Nonstatin Treatment**
**2016 ESC/EAS [10]** **(2017 update on PCSK9 mAb [65]** **)**	LDL-C < 70 mg/dL	LDL-C < 70 mg/dL (age > 40 years)	LDL-C < 100 mg/dL	If LDL-C above target, consider ezetimibe. If LDL-C > 100 mg/dL in ASCVD (+), also consider PCSK9 mAb. *
	**CHD (+)**		**CHD (−)**	**Nonstatin Treatment**
**2017 JAS** [12]	LDL-C <100 mg/dL (LDL-C <70 mg/dL in very high-risk patients)		LDL-C < 120 mg/dL (age 40–74 years)	If LDL-C above target, consider combination therapy (no specific drug indicated).
(b) Guidelines with statin intensity recommendations				
	**ASCVD (+) or LDL-C ≥ 190 mg/dL**		**ASCVD (−) and LDL-C 70–189 mg/dL**	**Nonstatin Treatment**
**2013 ACC/AHA [9**] **(2017 update on nonstatin treatment [66])**	High-intensity statin (moderate-intensity statin if age > 75 years)		Moderate-intensity statin (age 40–75 years)	If <50% LDL-C reduction, consider ezetimibe. If ASCVD (+), also consider PCSK9 mAb.
	**ASCVD (+)**		**ASCVD (−)**	**Nonstatin Treatment**
**2018 ADA** [2]	High-intensity statin		Moderate-intensity statin (age ≥ 40 years)	If LDL-C ≥ 70 mg/dL in ASCVD (+), consider ezetimibe or PCSK9 mAb.

AACE/ACE = American Association of Clinical Endocrinologists/American College of Endocrinology; ACC/AHA = American College of Cardiology/American Heart Association; ADA = American Diabetes Association; ASCVD = atherosclerotic cardiovascular disease; CAD = coronary artery disease; CVD = cardiovascular disease; ESC/EAS = European Society of Cardiology/European Atherosclerosis Society; JAS = Japan Atherosclerosis Society; LDL-C = low-density lipoprotein cholesterol, mAb = monoclonal antibody; PCSK9 = proprotein convertase subtilisin/kexin type 9; * LDL-C threshold, the starting value on which treatment decisions for a PCSK9 mAb are based, which is different from the LDL-C goal, the aim of therapeutic intervention.
