# Correction: Sugiyama, K.; et al. Management of Dyslipidemia in Type 2 Diabetes: Recent Advances in Nonstatin Treatment. *Diseases* 2018, *6*, 44

**DOI:** 10.3390/diseases6030061

**Published:** 2018-07-07

**Authors:** Kazutoshi Sugiyama, Yoshifumi Saisho

**Affiliations:** Division of Endocrinology, Metabolism, and Nephrology, Department of Internal Medicine, Keio University School of Medicine, 35 Shinanomachi, Shinjuku-ku, Tokyo 160-8582, Japan; ksugiyama@keio.jp

The authors wish to make the following changes to their paper [1]. In Table 1, in the last row, the authors reported rates of Neutralizing antibodies: 42% vs. 6% in ODYSSEY Outcomes. Actually, these are patient numbers and not percentages. Due to this fact, we would like to correct this data as 0.4% vs. 0.1% in Table 1. This correction has been made in both Table 1 and in the main text. 

Former Table 1:

New Table 1

The mistake in the main text

On page 5, Section 3.2.7, the sentence “In ODYSSEY Outcomes [19], neutralizing antibodies developed in 42% and 6% of patients in the alirocumab and placebo group, respectively,” should be replaced with “In ODYSSEY Outcomes [19], neutralizing antibodies developed in 0.4% and 0.1% of patients in the alirocumab and placebo group, respectively”.

The authors would like to apologize for any inconvenience caused to the readers by these changes.

## Figures and Tables

**Table 1:** Cardiovascular outcome trials of nonstatin drugs.

Variable	IMPROVE-IT [17]	FOURIER [18]	ODYSSEY Outcomes [19]
No. of patients	18,144	27,564	18,924
No. of patients with diabetes	4933 (27%)	11,031 (40%) [20]	5444 (29%)
Mean age (years)	64	63	58
Clinical characteristics	ACS within 10 days	ASCVD and LDL-C ≥70 mg/dL or non-HDL-C ≥100 mg/dL on statin	ACS within 12 months; LDL-C ≥70 mg/dL or non-HDL-C ≥100 mg/dL or ApoB ≥80 mg/dL on high-intensity statin
Intervention	Simvastatin 40 mg and ezetimibe 10 mg vs. simvastatin 40 mg	Evolocumab 140 mg q 2w or 420 mg q 4w vs. placebo	Alirocumab 75–150 mg q 2w vs. placebo
Primary endpoint	CV death, MI, stroke, hospitalization for UA, coronary revascularization	CV death, MI, stroke, hospitalization for UA, coronary revascularization	CHD death, MI, ischemic stroke, hospitalization for UA
Median f/u (years)	6	2.2	2.8
Achieved LDL-C (mg/dL)	53.7 vs. 69.5	30 vs. 92	53.3 vs. 101.4
Primary endpoint	32.7% vs. 34.7%; HR 0.936 (95% CI 0.89–0.99); *p* = 0.016	9.8% vs. 11.3%; HR 0.85 (95% CI 0.79–0.92); *p* < 0.001	9.5% vs. 11.1%; HR 0.85 (95% CI 0.78–0.93); *p* = 0.0003
3-point MACE (CV death, MI, stroke)	22.2% vs. 20.4%; HR 0.90 (95% CI 0.84–0.96); *p* = 0.003	5.9% vs. 7.4%; HR 0.80 (95% CI 0.73–0.88); *p*<0.001	10.3% vs. 11.9%; HR 0.86 (95% CI 0.79–0.93); *p* = 0.0003 *
CV death	6.8% vs. 6.9%; HR 1.00 (95% CI 0.89–1.13); *p* = 1.00	1.8% vs. 1.7%; HR 1.05 (95% CI 0.88–1.25); *p* = 0.62	2.5% vs. 2.9%; HR 0.88 (95% CI 0.74–1.05); *p* = 0.15
All-cause death	15.3% vs. 15.4%; HR 0.99 (95% CI 0.91–1.07); *p* = 0.78	3.2% vs. 3.1%; HR 1.04 (95% CI 0.91–1.19); *p* = 0.54	3.5% vs. 4.1%; HR 0.85 (95% CI 0.73–0.98); *p* = 0.026
Adverse events	Similar safety in both groups	Injection-site reactions: 2.1% vs. 1.6% Neutralizing antibodies: 0% in both groups	Injection site reactions: 3.8% vs. 2.1% Neutralizing antibodies: 42% vs. 6%

ACS = acute coronary syndrome; AMI = acute myocardial infarction; ApoB = apolipoprotein B; ASCVD = atherosclerotic cardiovascular disease; CHD = coronary heart disease; CI = confidence interval; CV = cardiovascular; FOURIER = Further Cardiovascular Outcomes Research with PCSK9 Inhibition in Subjects with Elevated Risk; HR = hazard ratio; HDL-C = high-density lipoprotein cholesterol; IMPROVE-IT = Improved Reduction of outcomes: Vytorin Efficacy International Trial; LDL-C = low-density lipoprotein cholesterol; MACE = major adverse cardiovascular events; MI = myocardial infarction; UA = unstable angina; * 3-point MACE for all-cause death, MI, stroke.

**Table 1:** Cardiovascular outcome trials of nonstatin drugs.

Variable	IMPROVE-IT [17]	FOURIER [18]	ODYSSEY Outcomes [19]
No. of patients	18,144	27,564	18,924
No. of patients with diabetes	4933 (27%)	11,031 (40%) [20]	5444 (29%)
Mean age (years)	64	63	58
Clinical characteristics	ACS within 10 days	ASCVD and LDL-C ≥70 mg/dL or non-HDL-C ≥100 mg/dL on statin	ACS within 12 months; LDL-C ≥70 mg/dL or non-HDL-C ≥100 mg/dL or ApoB ≥80 mg/dL on high-intensity statin
Intervention	Simvastatin 40 mg and ezetimibe 10 mg vs. simvastatin 40 mg	Evolocumab 140 mg q 2w or 420 mg q 4w vs. placebo	Alirocumab 75–150 mg q 2w vs. placebo
Primary endpoint	CV death, MI, stroke, hospitalization for UA, coronary revascularization	CV death, MI, stroke, hospitalization for UA, coronary revascularization	CHD death, MI, ischemic stroke, hospitalization for UA
Median f/u (years)	6	2.2	2.8
Achieved LDL-C (mg/dL)	53.7 vs. 69.5	30 vs. 92	53.3 vs. 101.4
Primary endpoint	32.7% vs. 34.7%; HR 0.936 (95% CI 0.89–0.99); *p* = 0.016	9.8% vs. 11.3%; HR 0.85 (95% CI 0.79–0.92); *p* < 0.001	9.5% vs. 11.1%; HR 0.85 (95% CI 0.78–0.93); *p* = 0.0003
3-point MACE (CV death, MI, stroke)	22.2% vs. 20.4%; HR 0.90 (95% CI 0.84–0.96); *p* = 0.003	5.9% vs. 7.4%; HR 0.80 (95% CI 0.73–0.88); *p*<0.001	10.3% vs. 11.9%; HR 0.86 (95% CI 0.79–0.93); *p* = 0.0003 *
CV death	6.8% vs. 6.9%; HR 1.00 (95% CI 0.89–1.13); *p* = 1.00	1.8% vs. 1.7%; HR 1.05 (95% CI 0.88–1.25); *p* = 0.62	2.5% vs. 2.9%; HR 0.88 (95% CI 0.74–1.05); *p* = 0.15
All-cause death	15.3% vs. 15.4%; HR 0.99 (95% CI 0.91–1.07); *p* = 0.78	3.2% vs. 3.1%; HR 1.04 (95% CI 0.91–1.19); *p* = 0.54	3.5% vs. 4.1%; HR 0.85 (95% CI 0.73–0.98); *p* = 0.026
Adverse events	Similar safety in both groups	Injection-site reactions: 2.1% vs. 1.6% Neutralizing antibodies: 0% in both groups	Injection site reactions: 3.8% vs. 2.1% Neutralizing antibodies: 0.4% vs. 0.1%

ACS = acute coronary syndrome; AMI = acute myocardial infarction; ApoB = apolipoprotein B; ASCVD = atherosclerotic cardiovascular disease; CHD = coronary heart disease; CI = confidence interval; CV = cardiovascular; FOURIER = Further Cardiovascular Outcomes Research with PCSK9 Inhibition in Subjects with Elevated Risk; HR = hazard ratio; HDL-C = high-density lipoprotein cholesterol; IMPROVE-IT = Improved Reduction of outcomes: Vytorin Efficacy International Trial; LDL-C = low-density lipoprotein cholesterol; MACE = major adverse cardiovascular events; MI = myocardial infarction; UA = unstable angina; * 3-point MACE for all-cause death, MI, stroke.
